# Sickness behavior in feverish children is independent of the severity of fever. An observational, multicenter study

**DOI:** 10.1371/journal.pone.0171670

**Published:** 2017-03-09

**Authors:** François Corrard, Christian Copin, Alain Wollner, Annie Elbez, Véronique Derkx, Stéphane Bechet, Corinne Levy, Michel Boucherat, Robert Cohen

**Affiliations:** 1 Association Clinique et Thérapeutique Infantile du Val-de-Marne. Saint-Maur-des-Fossés. France; 2 Association Française de Pédiatrie Ambulatoire, Chambéry, France; 3 Department of Microbiology, CHI Créteil, Créteil, France; TNO, NETHERLANDS

## Abstract

**Background. Objectives:**

Behavioral changes in a febrile child are usually considered to stem from the fever. We studied sickness behavior (SB) in terms of its clinical components and its relation to fever.

**Methods:**

This observational, multicenter study included children aged 6 months to 3 years who were either febrile (fever ≥12 hours, ≥ 39°C and ≥38°C at inclusion) or non-febrile and well. The child had to have been awake for the 2 hours preceding the consultation and cared for by the parent who brought him/her to the doctor. SB was evaluated according to 6 parameters over this 2-hour period: time spent playing, distance covered, time spent seeking comfort, time spent whining or crying, time spent in a state of irritation or of anger, most distorted facial expression. Two parameters were assessed for the 24-hour period preceding the consultation: time spent sleeping and appetite. The parent reported the degree of change in these parameters compared with the usual situation, using rating scales.

**Results:**

200 febrile children (most with nonspecific upper respiratory infections) and 200 non-febrile children were included. The mean values of the 8 parameters differed significantly (p<0.001) between the 2 groups and were independent of the height of fever at inclusion in the febrile children. In the study conditions, paracetamol failed to improve SB when the child was still feverish.

**Conclusion:**

The 8 parameters suggested that SB and fever are two independent manifestations that are activated simultaneously during an infection. This independence is in harmony with recommendations to treat the discomfort of SB and not the fever.

## Introduction

In the first years of life, children present acute illnesses, which contribute to the development of their immune system [1]. These infections may be accompanied by fever and behavioral changes, the latter being called sickness behavior (SB). SB is a set of changes in attitude and conduct and may be accompanied by discomfort. These changes may include decreased activity, lack of initiative, less liveliness, mood disorders with irritability, whimpering and greater tearfulness. The child may be harder to console, show more comfort-seeking behavior, reduced social interactions, less interest in the surroundings, have a distorted expression, a decreased appetite, and disturbed sleep [2].

Fever has long been held to be responsible for SB. Common expressions were “the child has difficulty tolerating the fever”, “discomfort of a fever”, “fever is uncomfortable”, “the child’s fever is causing him discomfort”. In fact, SB, like fever, results from the body’s reactions to invasion by a pathogen. It is triggered by proinflammatory cytokines produced by activated cells of the innate immune system in contact with specific pathogen-associated molecular patterns.

During an illness, SB may vary in presentation and intensity. When present, it may be of moderate expression without discomfort or marked and with a strong sense of malaise. Management of the sick child has greatly changed. Until the end of the last century, treatment was simple: use antipyretics to reduce the fever and also SB. The notion of discomfort in SB now guides treatment. French [3], Italian [4], American [5], British [6], and Canadian [7] recommendations for the management of the febrile child all have the same therapeutic aim: improved comfort, and no longer normalization of temperature. Better understanding of SB is essential for effective management of the febrile child.

Seven studies [8–14] have addressed SB, albeit with limitations (ill-defined, ill-quantified disparate criteria). The purpose of the present observational, multicenter, clinical study was twofold: to specify the clinical components of SB, by comparison of febrile and non-febrile children, and to study among febrile children the relationship between SB and the height of fever.

## Patients and methods

Private practice pediatricians included in this observational, multicenter study children aged from 6 months to 3 years with or without feverish illness. Inclusion criteria included febrile children with a rectal temperature ≥38°C on inclusion and a fever, lasting for ≥12 hours from the first measurement of temperature to the enrolment and which reach 39°C at least once.

Children with particularly painful illnesses, like acute otitis media or herpetic stomatitis, were excluded. Non-febrile controls also underwent measurement of rectal temperature, which was below 38°C. They were healthy, had come for a well-being visit, and were not on any ongoing treatment. Children with chronic diseases were excluded from both groups.

**Fig 1:**
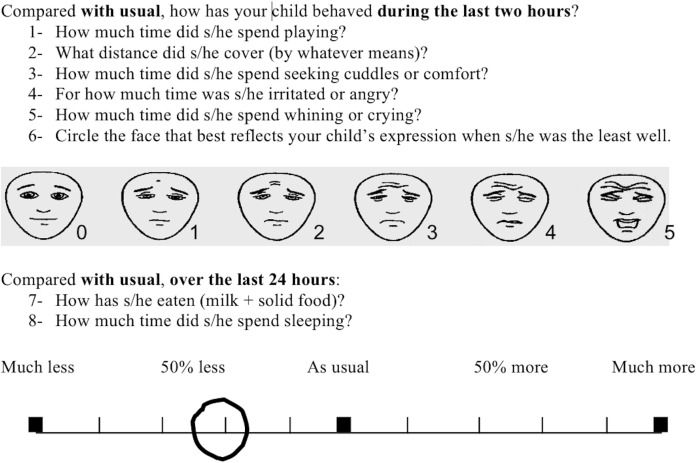
Questionnaire.

A poster in the playroom informed parents of the possibility of participating in this study, and that they were free to refuse. Parents were told that the study was anonymous and in no way affected the care their child received. After the medical examination, the inclusion criteria in both groups were checked, and the parents’ consent was collected before inclusion of their child. This study was approved by the ethics committee of the Hôpital Necker-Enfants Malades (Paris). The investigator then explained the questionnaire to the parents, who had to have a good understanding of French, and checked that they had understood. The parents were then asked to assess whether their child’s behavior had changed at all from the norm, and to quantify any change using 8 parameters (Fig 1), 6 during the last 2 hours and 2 over the last 24 hours. These parameters were selected from those used in studies of SB, those used to measure pediatric pain, and on the basis of the clinical experience of the study designers. Seven of these criteria were parameters that could be quantified: those relating to time, distance (if the child was old enough to move around), or amount of food ingested. Potential changes were evaluated using a visual analog scale with 10 graduations, indicating from left to right, at regular intervals, “not at all”, “50% less”, “as usual”, “50% more”, and “much more”.

The eighth criterion was facial expression, which the parents rated by indicating the most distorted facial expression during the 2 hours before the consultation, using a standard series of images of 6 faces expressing progressively increasing pain (Faces Pain Scale–Revised [FPS-R]) [15].

To allow sufficient observation time, the child had to be awake during the 2 hours preceding the consultation and the parent who brought the child had to be present beside the child during this time.

The questionnaire was tested before the study and modified slightly to improve parental understanding.

It was noted if the child had received antipyretic treatment, and which one (paracetamol or ibuprofen).

### Statistics

Each criterion was rated from 0 to 10 (0 to 6 for the faces). Feverish children were divided into 3 groups based on the severity of fever (38° to 38.5°C, 38.6°C to 39°C, 39.1°C and above) to study the relationship between SB and severity of fever. Data were entered by the software 4D (v2004) and analyzed using Stata SE 11.1 (Stata Corp., College Station, TX, USA). The Pearson chi-square test was used for univariate analyses and logistic regression for multivariate analysis.

## Results

Two-hundred febrile children and two-hundred non-febrile controls were enrolled from February 2011 to January 2012 by 20 private practice pediatricians in the Paris area. The 2 groups did not differ statistically in age (respectively 18.5 months ± 8.4 and 19.1 months ± 8.1) or sex (43.7% boys and 48% girls). In most cases (72%), the feverish condition was a nonspecific upper respiratory infection and 97% of the febrile children had received paracetamol or ibuprofen.

Comparison of SB parameters between febrile and non-febrile children.

**Fig 2:**
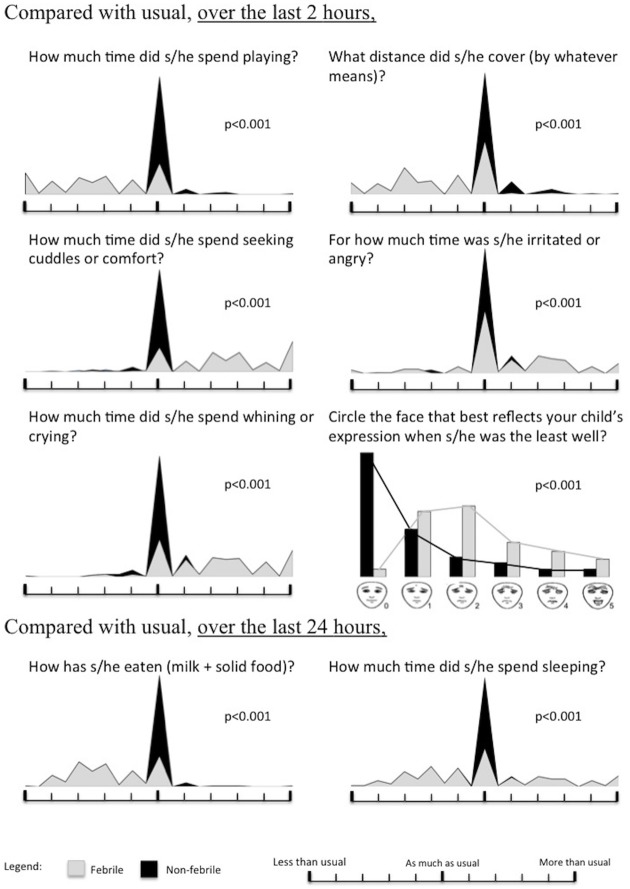
Univariate analysis. The mean scores for these 8 behavioral parameters are statistically very different, in the case of fever.

Univariate analysis (Fig 2) showed a very significant difference (p < 0.001) between the 2 groups in terms of the mean scores for the 8 behavioral parameters during the 2 hours before consultation: decrease in distance covered, decrease in time spent playing, increase in seeking cuddles or comfort, becoming irritated or angry, whining or crying, increase in distortion of facial expression. Over the 24 hours before the consultation, the decrease in the amount of food ingested was also statistically significantly different, and the time spent sleeping changed (increase or decrease). Multivariate analysis showed that time spent playing is important in characterizing SB (Table 1).

**Table 1:** Multivariate analysis.

	Adjusted OR [95% CI]	p
**Face score 1**	4.3 [1.5–12.9]	0.008
**Face score 2 or +**	6.2 [2.2–17.8]	0.001
**Cuddles, Comfort**	5.6 [2.6–12.0]	<0.001
**Eating**	12.5 [5.7–27.2]	<0.001
**Playing**	19.0 [7.8–46.4]	<0.001

Relative importance of the components of SB.

**Fig 3:**
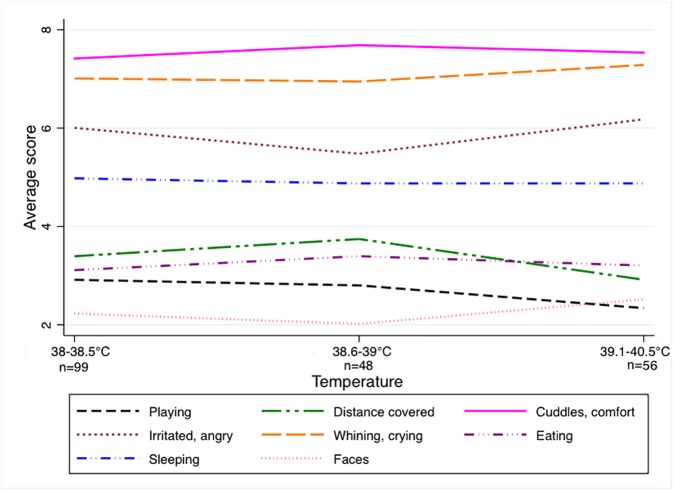
Independence of the 8 parameters with respect to the height of fever. The mean scores of the 8 parameters are independent of the height of fever at inclusion.

Variations in the 8 SB parameters in the 200 febrile children were independent of the severity of fever on inclusion. For each parameter, the distribution of the mean score for the 3 height of fever categories at inclusion (38° to 38.5°C, 38.6° to 39°C, 39.1°C and above) did not differ (Fig 3), in particular for the 6 parameters observed during the 2 hours preceding temperature measurement.

### Other properties of SB

The SB parameters were independent of age, sex and duration of fever, except for the youngest children (6–17 months), more of whom whined or cried than the older children (18–36 months) (p = 0.02). When the fever was prolonged (≥ 36 h), more children ate less than usual (p < 0.001).

## Discussion

To our knowledge, this is the first study to analyze behavioral differences between febrile and non-febrile children. When a fever was prolonged (≥12 h), and high (≥39°C) and when the child’s parents sought medical advice, the 8 parameters we used all discriminated well between the febrile children and non-febrile children. In the 2 hours before temperature measurement, the time spent playing and the distance covered were decreased in the febrile children. The time spent seeking cuddles and comfort, on the other hand, increased in febrile children, as did the length of periods of irritation, anger, or crying. The facial expression in the febrile children was more distorted. In the 24 hours before temperature measurement, appetite was decreased in the febrile children and time spent sleeping changed (increase or decrease). These 8 behavioral parameters are components of SB.

SB of the febrile child has been considered in 7 studies [8–14] (from 1991 to 2008). The primary outcome of these studies was decrease in temperature. SB was considered as a secondary outcome, except in the last study (2008) where it became the primary outcome along with antipyretic efficacy. The modalities for assessing SB in these studies had two points in common: recording of the parents’ perceptions and the use of several point scales, and important differences in criteria. Kramer et al [8] recorded mood, comfort, alertness, appetite and fluid intake. McIntyre and Hull [9] considered overall efficacy (which included temperature and behavior), irritability and clinical condition. Autret et al [10] assessed the child’s crying, facial expression, using the Children's Hospital of Eastern Ontario Pain Scale, general behavior and the child’s relief. Sarrell et al [11] assessed pain using the 27-item Non-Communicating Children's Pain Checklist. Autret-Leca et al [12] Kramer et al [13] rated overall efficacy. Hay et al [14] observed discomfort (according to mood and pain), activity, appetite and sleep.

This heterogeneity prompted us to check statistically 8 behavioral differences covering a large range of SB. One of these, time spent sleeping, is significantly different in the case of fever, but may decrease or increase. We felt that the use of references, such as time (how much time did he/she ….) or distance, could facilitate the quantification of parental perceptions.

In the 7 studies cited, SB was assessed without specifying the period of observation, which ranged from several hours to the entire duration of the febrile episode. Only Sarrell et al [11] mention a precise period of observation (10 min). We opted for a longer, precise period (2 hours).

During this observational period, the children served as their own controls, observed in their usual environment by a person who specifically knows them well. The temperature measurement was close to the observational period.

Ours is the first study to show a dissociation between SB and the severity of the fever, suggesting that these clinical manifestations are independent. Some children play as usual, even though their temperature is above 40°C. Others with a very low fever are affected more. The expression of SB also varies: some children are calm and just seem tired, whereas others appear distinctly uncomfortable.

The 8 signs could be the expression of a depressive episode: lack of energy, inappropriate reactions to daily ups and downs (tendency to become irritated, angry, to whine or cry easily, increased need for cuddles and comforting) and distortion of the facial expression, or even loss of appetite and altered sleeping time [16–18]. The 8 signs could be the expression of fatigue suspected when behavior is much improved by sleep unusual in terms of its length and when it occurs during the day. The 8 signs could also occasionally indicate behavior in response to pain [19–20]. But pain, which is not part of SB, on its own does not summarize the behavior of sick children. Moreover, to allow other causes of malaise to emerge, we excluded particularly painful illnesses (acute otitis media, herpetic stomatitis).

So, the change of behavior of the febrile child may reflect various causes—SB, pain, fatigue—at an age when there is no verbal expression, or not much. These causes are associated or not, which is generally impossible to distinguish. Regardless of this difficulty in identifying causes, what is important is to alleviate any discomfort. Signs suggestive of discomfort (tendency to become irritated or angry, to whine or cry, change in facial expression suggestive of pain) should be the target of treatment.

SB cannot be reduced to the observation of these 8 parameters. Its particularly rich variety of symptoms and signs covers other aspects, such as social activity, how the child interacts with others and with the environment, motivation to perform a task, behavioral flexibility, alertness of expression, and others [21]. We selected this limited number of criteria because they were easy for the parents to use and assess. Even though they represent a partial analysis of SB, changes in them were all independent of the severity of fever.

Our study suffers from some biases.

- Facial expressions were evaluated using the FPS-R, a series of 6 images of faces showing increasing degrees of pain (frowning, contraction of eyelids, wrinkled forehead, pinched mouth) [15]. The child is asked to point to the face that shows how much it hurts. Generally used and validated for self-assessment of pain from the age of 4, its presentation for such use calls for precautions when showing it to a child, by calling the extremes “no pain”, and “very much pain”. It is considered as the most reliable and reproducible known indicator of pain [19–20].

We used the FPS-R in a different setting, with children under 3 years old, without voicing the word “pain”, by asking the child’s parents to make the assessment by “circling the face closest to your child’s expression when he/she was worst over the last 2 hours”. This retrospective character of the study is regrettable, but only related to the 2 hours preceding inclusion and applied to both groups of children (febrile and non-febrile).

- This study is based on parents’ perceptions of their child’s behavior. Retrospective questioning only yields the changes recalled by the parents, which is different from a measurement. We tried to minimize this bias by choosing easily identifiable criteria and by codifying according to quantifiable values (time, distance, food intake, facial image) over an immediate and short period. In practice, the child is usually treated by the parents, in line with their perceptions. By taking this subjectivity into account, we approximate to real-life conditions.

The etiology of the fever of these children varied. Most children presented nonspecific upper respiratory tract infection. We minimized infections involving specific discomfort. Inclusions were mostly from spring to autumn, thus avoiding epidemic influenza and myalgia. Painful conditions (acute otitis media, mouth ulcers, gastroenteritis) were excluded.

Most of the febrile children (97%) had received antipyretics (87% paracetamol, 11% ibuprofen) since the beginning of the fever, some within a timeframe and at a dosage recommended at inclusion, others not. Some children (3%) did not receive treatment, but in all cases were febrile.

The independence of SB and fever suggests that they are expressions of 2 autonomous metabolic pathways that are activated simultaneously in febrile conditions. This is in accordance with current pathophysiological knowledge: innate immune cells, as polymorphonuclear leukocytes, monocytes, macrophages, recognize infectious microorganisms by means of specific receptors (toll-like receptors). They release proinflammatory cytokines, mainly interleukin I (IL-1), IL-6, and tumor necrosis factor alpha (TNFα), which may act directly on the brain or induce the enzyme cyclooxygenase 2 (COX-2) and the synthesis of prostaglandins of the E2 series ((PGE2). Fever is activated by PGE2, and SB depends on the effect of certain cytokines on the brain [16].

The depressive signs that occur in infectious diseases are related to the secretion of cytokines IL-1, IL-6 and TNFα [17, 22]. On injection of small doses of salmonella toxin, increases in IL-1 receptor antagonist (IL-1Ra, marker of increased IL-1), IL-6 and TNFα are associated with a more depressed mood compared with controls [23]. In H1N1 influenza, increase in IL-6 is associated with decreases in positive affect [24]. IL-1 is higher after birth in women with symptoms of depression than in women without depressive symptoms [25–26]. Meta-analyses [27–29] indicate that during depressive states there is an increase in the cytokines IL-1, IL-6 and TNFα. Genetic variation in IL-6 is associated with this expression of depression [30]. SB can therefore express a depressive episode induced by inflammatory cytokines. These depressive episodes differ from psychogenic depression as they are much shorter than the minimum of 2 weeks required by the DSM–IV-TR classification [31], and are rapidly reversible, probably because of the short half-lives of cytokines.

Fatigue, alone or associated with other depressive signs, is the first complaint reported during prolonged viral infections. There are links between inflammatory mediators, central neurotransmitters and fatigue [32–33]. The expression of fatigue is associated with interferon-gamma gene variation [30]. Loss of appetite is related to increases in IL-6 and TNFα, and vice versa [34].

Our clinical arguments for the independence of fever and SB are in line with pathophysiological understanding and point to the autonomy of these 2 metabolic pathways. In rats, there are differences in the mechanisms underlying activation of fever and SB. The vagal link between the immune system and the brain mediates behavioral depression, but not fever [35]. Vagotomy attenuates the behavioral but not the pyrogenic effects of IL-1 [36]. The receptor mechanisms that mediate the pyrogenic and behavioral effects of IL-1 are different [37]. An inhibitor of inflammatory gene expression acts differently on fever and SB [38].

The same inflammatory activation is responsible on the one hand for body temperature increase and on the other hand for SB, 2 phenomena that are concomitant and seem to be unconnected causally. Fever and SB may be 2 independent manifestations of innate immunity.

## Conclusion

Eight signs, chosen to describe SB broadly and accurately, all discriminated between febrile and non-febrile children. Variations in 6 of these signs, quantified over a 2-hour period before temperature measurement, were independent of the severity of fever. SB and fever seem to be clinically independent. This autonomy is suggestive of different modes of transmission of peripheral immune/inflammatory signals to the brain. This study backs up new treatment approaches to febrile children that take into account SB, particularly discomfort, rather than focusing on reducing fever. The 8 signs used here could therefore be useful in future studies of SB.

## Supporting information

S1 File Dataset. (XLS)
